# Detection of the *BRAF^V600E^* Mutation in Circulating Free Nucleic Acids as a Biomarker of Thyroid Cancer: A Review

**DOI:** 10.3390/jcm13185396

**Published:** 2024-09-12

**Authors:** Emilia Niedziela, Łukasz Niedziela, Aldona Kowalska, Artur Kowalik

**Affiliations:** 1 Department of Endocrinology, Holy Cross Cancer Center, 25-734 Kielce, Poland; emilia.niedziela@onkol.kielce.pl; 2 Collegium Medicum, Jan Kochanowski University, 25-317 Kielce, Poland; lukasz.niedziela@ujk.edu.pl; 3 Department of Molecular Diagnostics, Holy Cross Cancer Center, 25-734 Kielce, Poland; artur.kowalik@onkol.kielce.pl; 4 Division of Medical Biology, Institute of Biology, Jan Kochanowski University, 25-406 Kielce, Poland

**Keywords:** thyroid cancer, liquid biopsy, ctDNA, *BRAF^V600E^*, molecular diagnostics

## Abstract

**Background**: Liquid biopsy is a method that could potentially improve the management of thyroid cancer (TC) by enabling the detection of circulating tumor DNA and RNA (ctDNA, ctRNA). The *BRAF^V600E^* mutation appears to be the most representative example of a biomarker in liquid biopsy, as it is the most specific mutation for TC and a target for molecular therapeutics. The aim of this review is to summarize the available data on the detection of the *BRAF^V600E^* mutation in liquid biopsy in patients with TC. **Methods**: A comprehensive analysis of the available literature on the detection of the *BRAF^V600E^* mutation in liquid biopsy in TC was performed. Thirty-three papers meeting the inclusion criteria were selected after full-text evaluation. **Results**: Eleven papers discussed correlations between *BRAF* mutation and clinicopathological characteristics. Nine studies tested the utility of *BRAF^V600E^* detection in the assessment of residual or recurrent disease. Seven studies investigated *BRAF*-mutated circulating tumor nucleic acids (ctNA) as a marker of response to targeted therapy. In seven studies the method did not detect the *BRAF^V600E^* mutation. **Conclusions**: This review shows the potential of *BRAF^V600E^*-mutated ctNA detection in monitoring disease progression, particularly in advanced TC. The diagnostic value of *BRAF^V600E^*-mutated ctNA detection appears to be limited to advanced TC. The choice of the molecular method (quantitative PCR [qPCR], droplet digital polymerase chain reaction [ddPCR], and next-generation sequencing [NGS]) should be made based on the turnaround time, sensitivity of the test, and the clinical indications. Despite the promising outcomes of some studies, there is a need to verify these results on larger cohorts and to unify the molecular methods.

## 1. Introduction

Thyroid cancer (TC) is the most common endocrine malignancy worldwide. In 2022, the World Health Organization published an updated classification of tumors that divided thyroid neoplasms into new categories according to pathophysiological and molecular features [1]. There are five main types of TC: papillary (PTC), follicular (FTC), and oncocytic (OCT), which are described together as differentiated TCs (DTCs), medullary TC (MTC), and anaplastic TC (ATC). DTCs account for approximately 90% of all TC cases [2,3]. Although guidelines for the management of TC are well-established, detection and assessment of mutations and chromosomal rearrangements show great potential to address problems related to the diagnosis, treatment, and follow up of thyroid lesions [4,5].

**Figure 1:**
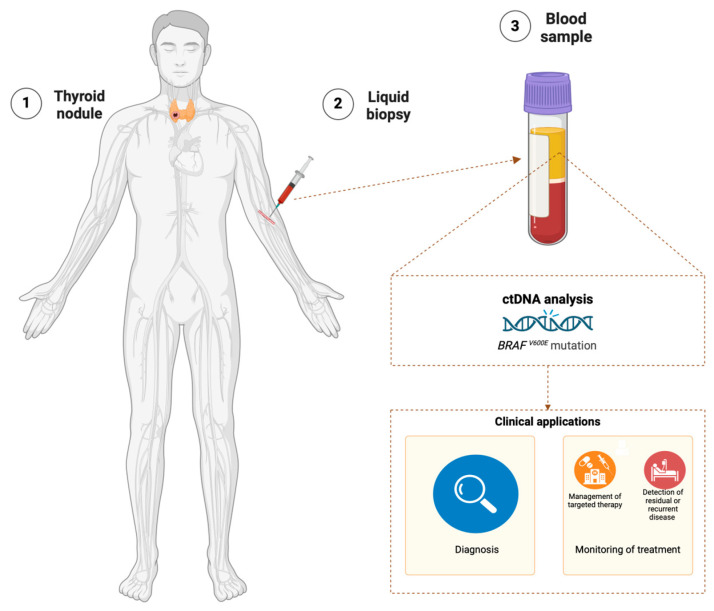
Detection of *BRAF^V600E^* mutation in the plasma of patients with TC. Created with BioRender.com (accessed on 17 August 2024).

One promising new method that may improve the diagnosis of TC is liquid biopsy, which detects the presence of circulating tumor cells (CTCs) and circulating extracellular nucleic acids, including circulating free DNA (cfDNA) and circulating free RNA (cfRNA), in biological fluids (Figure 1). Liquid biopsy provides genetic and epigenetic information about tumors in a non-invasive, patient-acceptable, and repeatable manner. Although liquid biopsy is an established modality, e.g., in lung cancer, its utility in the management of TC in clinical practice remains to be determined [6].

Detecting cancer-derived nucleic acids requires the correct selection of a mutation or epigenetic alteration that is highly specific for cancer. Due to their high prevalence, mutations in the *BRAF* proto-oncogene have been tested as potential markers for circulating tumor nucleic acids in patients with TC. The aim of this review was to summarize current data on the detection of the *BRAF^V600E^* mutation in liquid biopsy as a marker for TC diagnosis and monitoring.

### 1.1. BRAF^V600E^ Mutation Role in Oncogenesis

The influence of molecular alterations on the histology and clinical behavior of follicular cell-derived cancers is reflected in the classification of DTCs into *BRAF*-like and *RAS*-like, in addition to a third group of non-*BRAF*-/non-*RAS*-like tumors [7,8]. In a Cancer Genome Atlas study that analyzed the molecular profile of almost 500 cases of PTC, *BRAF* mutations accounted for 61.7% of all mutations; most mutations were substitutions at codon V600 [9]. The frequency of *BRAF^V600E^*-positive PTCs is increasing [10].

**Figure 2:**
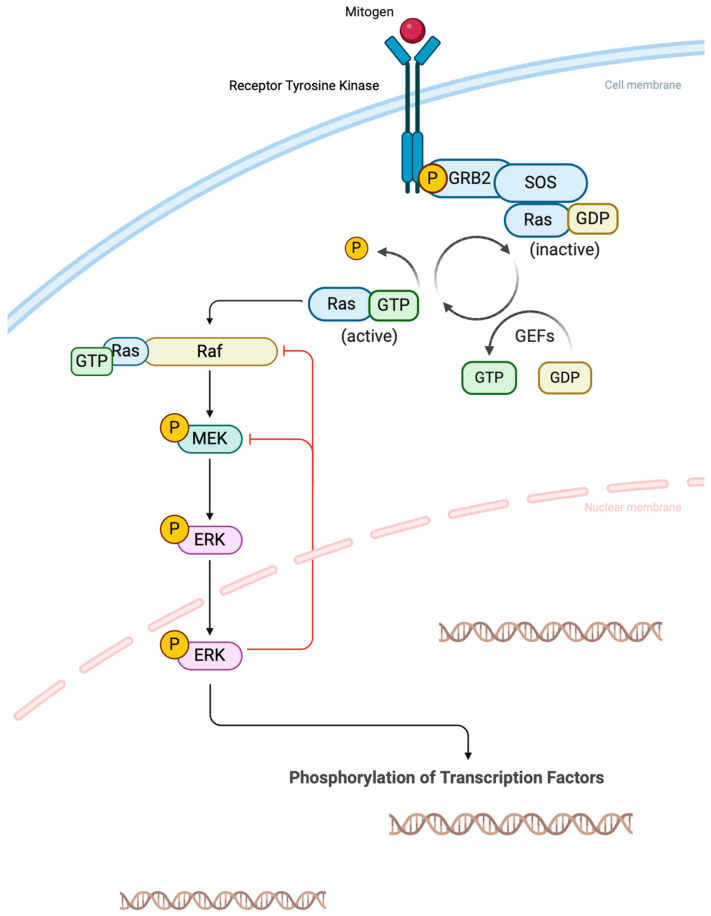
Schematic representation of the conventional mitogen-activated protein kinase (MAPK) pathway. Mitogens stimulate the receptor tyrosine kinase, and activation of downstream kinases results in the phosphorylation of transcription factors responsible for cell growth, proliferation, and survival. Created with BioRender.com (accessed on 17 August 2024).

The transversion of thymidine to adenosine at exon 15 nucleotide 1799 (T1799A) of the *BRAF* gene results in the substitution of valine for glutamic acid at position 600 (*BRAF^V600E^*). The *BRAF* gene encodes the serine/threonine-protein kinase B-Raf, a signaling molecule in the mitogen-activated protein kinase (MAPK) pathway. Cancers associated with the *BRAF^V600E^* mutation do not respond to negative feedback from the extracellular signal-regulated kinase (ERK) to the mutant Raf monomer; this causes increased MAPK signaling, which leads to increased transcriptional efficiency and MEK/ERK-dependent transformation (Figure 2). *RAS*- and *RTK*-driven fusion tumors signal through Raf dimers that respond to ERK negative feedback, resulting in decreased MAPK signaling [11].

The *BRAF^V600E^* mutation is the most common driver mutation in PTC and is associated with less differentiated histologic subtypes, including classical histology, as well as more aggressive tall and columnar cell subtypes [1]. Poorly differentiated cells or highly differentiated cells can be present in a tumor lesion composed of undifferentiated cells, indicating the gradual transformation of DTC into ATC [12]. The high incidence of the *BRAF^V600E^* mutation in ATC suggests that the gradual acquisition of other mutations leads to greater cell dedifferentiation [13]. In *BRAF*-positive cancers, key genes involved in iodine uptake and metabolism are downregulated, consistent with the higher frequency of radioactive iodine resistant (RAIR) *BRAF*-positive metastatic lesions [14,15]. *BRAF^V600E^*-positive TCs include subtypes with different molecular profiles and degrees of differentiation, which is an important consideration regarding the clinical outcomes of patients with the *BRAF^V600E^* mutation [7,14,15].

### 1.2. Circulating Tumor DNA (ctDNA)

Advances in molecular biology techniques have enabled the detection and application of circulating free nucleic acids as carriers of molecular information. In normal tissues, DNA is mostly removed via apoptosis, and only a small amount, mostly fragments of 185–200 bp, enters the bloodstream [16,17]. Pathological processes lead to an abnormal release of genetic material. cfDNA is not necessarily of tumor origin, whereas circulating tumor DNA (ctDNA) contains mutations specific for neoplasms. ctDNA has a short half-life of almost 2 h, which makes it a good candidate for serial assays.

## 2. Materials and Methods

**Figure 3:**
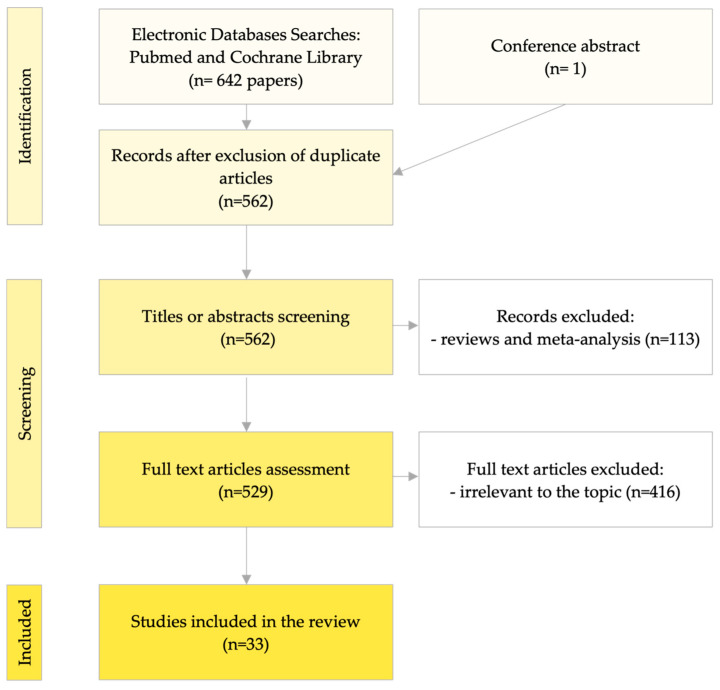
PRISMA 2020 flow diagram of the article selection process [18].

A comprehensive analysis of the available literature was performed to present a reliable summary of current data. The Pubmed and Cochrane Library databases were searched using combinations of keywords and abbreviations as well as spelling variations such as the following: ‘*BRAF^V600E^* mutation’, ‘circulating free DNA’, ‘circulating tumor DNA’, ‘circulating nucleic acids’, ‘liquid biopsy’, ‘plasma’, ‘blood’, ‘serum’, and ‘thyroid cancer’. The electronic search was supplemented by checking the reference lists of selected articles. Because studies that include large groups of patients often provide detailed descriptions of a few patients, case reports were also included, and there were no limitations in the number of patients. Studies published before 2000, studies in a language other than English, reviews, and meta-analyses were excluded from the review. The data extracted from the articles included the year of publication, number of patients, TC histology, stage, nucleic acid type, the analytical modality and its sensitivity, specificity, concordance with the tissue, and main findings. These values referred to molecular assays of tissue as the gold standard. Of the 562 papers screened, 33 that met the inclusion criteria were selected after a full-text assessment (Figure 3). One paper from a congress report was included. The total number of patients was 3369. The results of the review are presented in descriptive form because of the variety of study designs and analytical methods used. To the best of our knowledge, there is currently no review on *BRAF^V600E^* detection in liquid biopsy in patients with TC.

## 3. Results

### 3.1. Mutation Analysis

The techniques currently available for the detection and analysis of ctDNA are based on selective amplification [e.g., quantitative PCR (qPCR), droplet digital polymerase chain reaction (ddPCR)] and next-generation sequencing (NGS) (Table 1).

Most ctDNA testing is based on qPCR because it is cost-effective and allows for a real-time quantitative evaluation of samples. This method provides an accurate estimation of the DNA fragments present prior to the reaction and can target a selected mutation, which is useful for obtaining information on possible drug targets.

A ddPCR is used to detect point mutations in ctDNA at low allele fractions. The method is based on dispersing a DNA sample into thousands to millions of droplets in a water–oil emulsion. Each droplet contains a single mutant or wild-type DNA strand, which can be distinguished via flow cytometry using TaqMan-based fluorescent probes. The main advantages of this method are low costs and high sensitivity. However, similar to qPCR, it can only detect known variants, and the number of variants that can be tested in a single reaction is limited [26].

NGS provides a comprehensive profile of molecular alterations in ctDNA via simultaneously analyzing millions of DNA fragments, and it can identify mutations with an allele frequency of less than 0.01% depending on the method used. Bioinformatics tools and data analysis applications are used for comparisons with a reference sequence and identification of pathogenic variants [27]. NGS is particularly useful for the comparative assessment of tumor tissues and circulating nucleic acids.

An improvement in NGS technology is molecular barcoding, which involves attaching short unique nucleotide sequences to DNA fragments during library preparation. By enabling the identification of the origin of each DNA strand, it allows the analysis of multiple samples simultaneously, increasing the detection of rare variants through reducing PCR or sequencing errors (false positive variant) and removing PCR duplicates [23,24].

The largest number (13) of studies reviewed used qPCR. There is a clear trend in recent years toward the use of ddPCR (seven studies) and NGS (seven studies). Variations in parameters such as sensitivity, specificity, and concordance were observed even among studies that used the same method (Table 2). However, differences in the clinical and pathological characterization of the studied groups may have affected the results (Table 3).

### 3.2. Potential Applications of BRAF^V600E^ Mutation in Liquid Biopsy

#### 3.2.1. Diagnosis

The narrowing of ultrasound criteria for identifying high-risk lesions resulted in a 0.5% yearly decrease in the incidences of TC between 2010 and 2019. However, inconclusive results of fine needle aspiration biopsy (FNAB) often lead to repetitive procedures and unnecessary thyroid surgeries [61]. Therefore, identifying accurate diagnostic methods for TC is critical.

##### Correlation with Clinicopathological Features

Correlations between *BRAF* mutation and clinicopathological characteristics were reported in 11 studies. The presence of *BRAF*-mutated ctDNA was correlated with higher stage, extrathyroidal extension, lymph node metastasis (LNM), and distant metastasis.

Five studies reported an association between ctDNA *BRAF* positivity and LNM in patients with PTC (Chuang et al., Kim et al., Lubitz et al., 2016, Jensen et al., Khatami et al.) [29,35,36,46,47]. In a study by Chuang et al., out of 14 patients diagnosed with PTC using pathology, 5 had the *BRAF^V600E^* mutation in tumor tissue. In three patients (60%), the *BRAF^V600E^* mutation was detected in matching sera collected before thyroid surgery, and two PTC patients had LNM [29].

Four studies reported a correlation between *BRAF^V600E^* status and extrathyroidal extension (Lubitz et al., 2018, Jensen et al., Sato et al., Patel et al.) [44,46,55,56]. Sato et al. presented data from 16 patients with *BRAF^V600E^* mutation in the primary tumor. Five (31%) showed positive *BRAF^V600E^* ctDNA in the presurgical analysis. Positive *BRAF^V600E^* status in ctDNA correlated with extrathyroidal extension and a high ratio of *BRAF^V600E^* alleles to total *BRAF* alleles in the primary tumor [55]. A 2018 study by Lubitz et al. used reverse transcription analysis to detect the *BRAF^V600E^* mutation in circulating tumor RNA (ctRNA) from 54 patients who underwent initial surgery for PTC and found a significant correlation between preoperative *BRAF^V600E^* ctRNA levels and extrathyroidal extension [44]. Patel et al. analyzed the ctDNA from 109 patients who underwent partial or total thyroidectomy using pre- and postoperative blood samples. Positive *BRAF* status in ctDNA was detected only in patients with classical PTC (*n* = 15) and was associated with higher stage (T3–4) and extrathyroidal extension [56].

Two studies reported a higher incidence of distant metastasis and advanced disease in treatment-naive patients with detectable *BRAF^V600E^* ctDNA (Kim et al., Jensen et al.) [35,46]. In a study by Kim et al., *BRAF*-mutated ctDNA was detected in three patients with lymph node and lung metastases [35]. Jensen et al. assessed the frequency of *BRAF^V600E^* mutation in ctDNA using a combination of ddPCR and co-amplification at lower denaturation temperature PCR [(COLD)PCR] in the plasma of 57 patients with *BRAF^V600E^*-positive PTC. *BRAF*-mutated ctDNA was detected in 42.1% of the samples and was positively correlated with tumor size, multifocal disease, gross extrathyroidal extension, and the presence of lung micrometastases. The prevalence of *BRAF*-mutated ctDNA was higher among high-risk PTCs according to the ATA (12/16, 75.0%). Patients with positive *BRAF*-mutated ctDNA had an increased risk in poor responses to treatment, including those with low-risk PTCs. The results of this study indicate that the detection of *BRAF*-mutated ctDNA may be a risk factor for poorer prognosis. One advantage of this study was the use of (COLD)PCR, which increased the sensitivity for detecting low-frequency mutant alleles 100-fold over other methods. The use of digital PCR alone showed a sensitivity of 14% in a group of mostly high-risk cancers [46].

Li et al. did not find an association between mutations in ctDNA and clinicopathological features [45]. Gouda et al. showed that patients with a wild-type (wt) allele in ctDNA and tissue had a shorter overall survival than those with detectable *BRAF* mutation [57]. A recent study by Tarasova et al. on plasma NGS testing detected the *BRAF^V600E^* mutation in 27.2% of ATCs, 35.7% of PTCs, and in none of the other types of TC [60].

##### Risk of False-Positive Results

False-positive outcomes were reported in several studies (Pupilli et al., Lubitz et al., 2016, Lubitz et al., 2018, Li et al., Wei et al., 2020, Patel et al., Gouda et al., Wei et al., 2022) [31,36,44,45,48,56,57,58]. The use of high sensitivity methods without established cut-off points and the presence of a different, undiagnosed neoplasm with a high prevalence of *BRAF* mutations (e.g., melanoma and colorectal cancer) may contribute to false positivity. Wei et al. reported that the mutation was detected in one of five benign thyroid nodules (TN) and two ctDNA samples. An analysis of 10 plasma samples from randomly selected patients with various cancers detected the *BRAF^V600E^* mutation in three cases (oropharyngeal cancer, T-lymphoblastoma, and gastric cancer) [48]. Lubitz et al. reported a patient with the detectable *BRAF* mutant ctRNA with a benign thyroid lesion, who was further diagnosed with melanoma [36].

Pupilli et al. showed that the mutation is detectable in the cfDNA of patients with benign histology, although the rate of mutated cfDNA was significantly lower in this group than in patients with confirmed TC [31]. Li et al. reported that in three patients (38.46%) with negative *BRAF^V600E^* mutation status in tumor tissue, the mutation was detected in cfDNA [45]. Wei et al. detected the *BRAF^V600E^* mutation in the plasma of two patients with TN [58], whereas Lubitz et al. detected baseline *BRAF^V600E^* signals in *BRAF* wt TC as well as in benign lesions [44].

One reason for the discordant results between tissue and cfDNA assays may be the determination of *BRAF* mutation status only in the predominant tumor nodule, considering the tendency for heterogeneous molecular profiles and the multifocality of PTC [8,62]. Assessment of the mutation in tissue samples may misrepresent the disease status.

Fibbi et al. described the case of a 49-year-old patient diagnosed with MTC, PTC, and cutaneous melanoma. *BRAF^V600E^* mutation was detected in PTC and melanoma tissues, and the positivity rate of mutant BRAF was higher in presurgical ctDNA than in samples collected after the treatment for PTC and melanoma (99.8% vs. 0.07%). However, there were no data on the decrease in *BRAF^V600E^* levels after thyroid surgery. This case shows that the co-occurrence of different cancers with *BRAF^V600E^* mutation may be a significant limitation of the method, and having detailed information about the clinical status (radiology, biochemical tests) of the patient could be helpful to interpret the results of the liquid biopsy [34].

##### Risk of False-Negative Results

In contrast to the results presented above, some studies described the low accuracy of assays for the detection of *BRAF* in ctDNA. Kwak et al. reported that in 94 patients with *BRAF* mutation detected via a FNAB, mutant ctDNA was not detected in any of the presurgical samples [33]. In 2004, Vdovichenko et al. published the results of a study on *BRAF^V600E^*-mutated ctDNA in patients with various cancers. In patients with thyroid tumors (*n* = 6) of stage 0–1, the *BRAF* mutation was not detected in tissue or in ctDNA [28]. Lupo et al. analyzed the plasma of 56 patients with thyroid nodules and did not detect the *BRAF* mutation in any of the samples. However, this study was limited by the lack of histopathological verification of the FNAB results in some patients and the absence of data on *BRAF* status in tumor tissue [39]. In a study by Condello et al., both qPCR and ddPCR failed to detect *BRAF^V600E^*-mutated ctDNA in 22 patients diagnosed with *BRAF*-positive PTC. Although this study did not validate the ctDNA assessment methods and lacked standard positive controls, data obtained using positive and negative control samples from *BRAF*-positive colorectal cancer showed 100% concordance [42]. A 2021 study by Suh et al. showed that ctDNA was negative for the *BRAF* mutation in all patients with *BRAF*-mutated PTC. However, the study had a high percentage of failed tests due to repetitive freezing and thawing of extracted cfDNA, which may have affected the results [53]. Cao et al. failed to detect *BRAF*-mutated ctDNA in all plasma samples from patients with *BRAF*-positive PTC. The patients included in the study had low stage disease, which may account for the reduced release of ctDNA into the blood [49]. Zane et al. was unable to detect the *BRAF* mutation in ctDNA from 86 patients with PTC because of difficulties in recovering the material [32].

In 2020, Lan et al. explored the prevalence of molecular alterations in metastatic PTC. Although the *BRAF^V600E^* mutation was the most common mutation detected in tumor tissues (73%), the sensitivity of the method used for ctDNA was 4.5%. Patients without metastases showed no driver mutations in ctDNA. The *BRAF^V600E^* mutation was more common in small locoregional tumors, which might explain the low rate of detection in the plasma [52].

#### 3.2.2. Further Management of TC

Another potential application of *BRAF^V600E^* mutation detection is for disease monitoring, especially in situations where the conventional marker thyroglobulin (Tg) is not useful. Detecting the presence of the *BRAF^V600E^* mutation in the blood can be used to identify possible recurrence or persistence of the disease in patients with non-Tg-secreting TC, anti-Tg antibodies, after lobectomy, or in those with suspected metastases to sites that cannot be accessed via biopsy. Given the increased availability in molecular targeted therapies, ctDNA analysis can be used to design personalized treatments for patients with unresectable or metastatic tumors.

##### Detection of Residual or Recurrent Disease

Detection of *BRAF^V600E^* in postoperative plasma may indicate active disease and lead to more detailed follow-ups of selected patients. Nine studies described the utility of *BRAF^V600E^* detection in the assessment of residual or recurrent disease.

The Almubarak study correlated *BRAF*-mutated ctDNA levels with the presence of residual disease. The sensitivity and specificity of ctDNA levels for predicting disease progression were higher than those of Tg (sensitivity, 86% vs. 78%; specificity, 90% vs. 65%). Median plasma ctDNA levels were significantly higher in metastatic than in non-metastatic disease. In addition, total plasma cfDNA levels were significantly lower in patients with persistent disease than in patients without evident disease, although the authors were unable to explain this phenomenon. Combining the Tg assay with ctDNA detection may increase the sensitivity for detecting residual disease, given the different limiting factors of each assay [51].

Dutta et al. reported that three patients with *BRAF*-mutated ctDNA in the postoperative follow-up had persistent disease and LNM [59].

A study by Pupilli et al. showed that patients who remained strongly *BRAF* mutation-positive in postoperative ctDNA (*n* = 2) had a high risk of persistent disease, which was due to elevated Tg in one and incomplete resection in the other [31].

Sato et al. reported that the only patient with *BRAF*-mutated ctDNA detectable after surgery developed LNM 6 months after a thyroidectomy [55].

In a study by Patel et al., the 13 patients with *BRAF*-positive cancers who underwent surveillance showed reduced postoperative ctDNA levels, and *BRAF* ctDNA was undetectable in 12 cases. The only *BRAF* ctDNA-positive patient at the postoperative surveillance had incomplete tumor resection and suspicion of persistent disease [56].

In patients with metastatic TC, Gouda et al. showed that there was no qualitative or quantitative correlation between Tg and *BRAF* in ctDNA [57].

Sandulache et al. reported that in two patients with *BRAF^V600E^*-positive ATC without active disease on imaging, and in one during systemic treatment, *BRAF* mutation was not detected in ctDNA. The authors highlighted that an additional advantage of liquid biopsy, which was noted for other genes, is that it provides information on clonal tumor growth, especially after primary systemic treatment has been initiated. It may indicate the molecular evolution of the tumor, thereby allowing modification of the therapy without the need for invasive tissue sampling, compared to the minimally invasive process of simple blood collection. Tissue collection on cell blocks or during FNAB may yield false-negative results due to the tendency for necrosis and the heterogeneity of ATC [40].

In 2018, Allin et al. published the results of a study evaluating ctDNA as a biomarker for monitoring advanced TC. The *BRAF^V600E^* mutation accounted for 61% (13/21) of all detected mutations in PTC tissues and for 7% (1/14) in MTC. The occurrence of the identified mutations in ctDNA was then analyzed by ddPCR at sequential timepoints. In one patient with a present anti-Tg, detection of *BRAF*-mutated ctDNA preceded detection of disease progression on imaging scans. A similar observation was made in a group of PTC patients treated with targeted therapies, in which changes in ctDNA levels were a better indicator of the efficacy of the therapy than conventional markers. The results of this study support that an extensive analysis of the range of mutations in tumor tissue using ctDNA, especially when planning targeted therapy, is valuable to monitor the course of the disease non-invasively in patients with advanced TC [41].

In a study by Cradic et al. among 42 patients with positive *BRAF^V600E^* status in tissue, 8 (19%) had a detectable mutation in ctDNA. Detection of ctDNA correlated with the presence of persistent disease or disease recurrence at the time of blood draw. Although *BRAF* ctDNA was a valuable marker of tumor burden and disease status, it did not show superiority over Tg levels [30].

##### Management of Targeted Therapy

In up to 23% of DTC cases, distant metastases are present at the time of diagnosis or detected during the follow up with primary or acquired RAI resistance [63,64]. ATC is resistant to RAI. Standard chemotherapy is ineffective in most cases, and molecular targeted therapy is thus a promising therapeutic option for patients with RAI-resistant TC.12 The identification of molecular targets resulted in the approval of new drugs for the treatment of *BRAF*-mutated PTC, including dabrafenib, trametinib, and vemurafenib [12,65,66]. ATCs with the *BRAF^V600E^* mutation can receive combination therapy with *BRAF* and the MEK inhibitors dabrafenib and trametinib [67,68]. Clinical trials of combination therapy with BRAF-inhibiting drugs (BRAFi) and MEK-inhibiting drugs (MEKi) in patients with other *BRAF^V600E^*-mutated TC are ongoing [69].

Liquid biopsy may be particularly valuable in cases in which it is not possible to obtain tissue material to verify whether the patient is eligible for targeted therapy. Patients are often treated surgically at centers that do not offer oncological follow-ups, which may result in the lack of material suitable for molecular testing. In addition, ctDNA analysis provides information about intra- and inter-tumor heterogeneity, which would be difficult to achieve with tissue biopsy [70]. It can also be serially assessed at set intervals to analyze changes during treatment [71].

Seven studies tested *BRAF*-mutated ctDNA as a marker of tumor molecular status and response to targeted therapy.

Qin et al. investigated the clinical utility of ctDNA in patients with ATC. Only the *PIK3CA* mutation in ctDNA was associated with worse overall survival regardless of treatment and the presence of *BRAF* mutation. These results support the analysis of multiple mutations in ctDNA to improve prognosis prediction, as well as combination therapy with the PIK3CA inhibitor drug alpelisib, which is currently only available for the treatment of advanced breast cancer. The study showed that ctDNA is a reliable source of information on the molecular landscape of ATC, which has implications for tailoring targeted treatment [54].

In 2020, Cabanillas et al. presented case reports of patients with TC treated with BRAFi who experienced progression associated with acquisition of *RAS* mutations. In one patient with ATC, liquid biopsy was used to detect the *BRAF^V600E^* mutation before combination treatment with BRAFi and MEKi because the tumor biopsy material was non-diagnostic. In one patient with recurrent and metastatic PTC, the presence of the *BRAF^V600E^* mutation was confirmed using NGS in both the tumor and liquid biopsy. In one patient with metastatic ATC, the *BRAF* mutation was detected in ctDNA after progression on targeted therapy, which was consistent with the tissue analysis results [50].

Lubitz et al. showed that ctRNA is a valuable source for biochemical monitoring of advanced TC, especially in cases with no Tg production or with anti-Tg elevation. The study showed that *BRAF^V600E^* levels decreased significantly shortly after the initiation of targeted therapy and corresponded to the radiographic assessment of partial response (PR) or stable disease (SD). Of three patients with increased ctRNA levels detected during the follow up, one experienced disease progression and the other two maintained SD status. In the group with RAIR metastatic TC, *BRAF* levels were positive in four patients despite the negative status of the primary tumor tissue, which may reflect tumor heterogeneity or the development of a new clone of cells responsible for disease progression. The results of the study indicate that detection of mutations in advanced cancer may provide an alternative to conventional markers for detecting disease progression [44].

A study by Iyer et al. evaluated the utility of *BRAF^V600E^* mutation as a biomarker for the management of ATC. In a group of 16 monitored patients, a comparison of 36 imaging scans with *BRAF*-mutated ctDNA levels showed 75% concordance between ctDNA changes and response to therapy. The ctDNA levels correlated with decreased tumor burden in 94% of patients and with tumor growth in 47% of patients; SD was associated with stable ctDNA levels in all cases. In 12/17 (71%) of the samples collected between scans, ctDNA concentrations were predictive of treatment response. The earliest change in ctDNA levels was detected 1 day after surgical treatment and 2 weeks after the initiation of BRAFi. Three patients had baseline false-negative ctDNA results. In this group, ctDNA levels remained low in all patients, consistent with a favorable imaging response to BRAFi treatment [43].

Konda et al. compared *BRAF^V600E^* ctDNA levels with the results of the imaging evaluation of treatment response in 20 *BRAF^V600E^*-positive RAIR DTC patients enrolled in a randomized multicenter phase II study of dabrafenib versus dabrafenib plus trametinib. Seven patients had detectable levels of *BRAF^V600E^* at baseline, and all of them had undetectable *BRAF^V600E^* levels after 2 months of treatment. Five patients achieved PR during treatment, and two showed SD as the best response. In a comparison of Tg levels with ctDNA levels, two patients showed a 2-fold increase in Tg during the PR period with undetectable ctDNA. At the end of the follow up, in 3/4 patients excluded from the study due to progressive disease, *BRAF^V600E^* ctDNA became detectable at the time of progression, and in one of these patients this increase preceded progression on imaging studies [38].

The usefulness of *BRAF^V600E^* mutation detection in ctDNA in advanced cancers was investigated in a study by Janku et al. Eight patients had detectable *BRAF* mutation in tumor tissue. In five of these patients, the *BRAF^V600E^* mutation was also detected in ctDNA. Two patients with TC had ctDNA evaluation before initiation of BRAFi treatment and during therapy. The changes in the rate of mutant ctDNA were consistent with the changes in Tg concentration, and both parameters decreased after the start of therapy [37].

Detection of *BRAF^V600E^* mutation in a study by Sandulache et al. showed 100% concordance between tissue and plasma in patients with ATC prior to adjuvant treatment. This study highlighted the significantly shorter turnaround time of ctDNA analysis compared with conventional mutation testing in tumor tissue, which is an argument supporting the use of ctDNA for fast assessment before introducing targeted treatment [40].

## 4. Discussion

*BRAF^V600E^* mutation is specific to PTC and ATC, and its detection in cfNA has been tested as a marker for those malignancies. However, the results of the studies included in this review were inconsistent. The differences can be mostly attributed to preanalytical and analytical issues, the methods used, and the patients analyzed. *BRAF* mutation is a novel marker that lacks extensive validation, and it should therefore be used as a follow-up marker in cases with proven positive *BRAF* mutation status in tumor tissues rather than as a single biomarker. Determining the threshold for functional sensitivity is important to exclude false-positive results caused by low or absent *BRAF^V600E^*. Additional data are needed to establish cut-off points to minimize the risk of false-negative results. The mutated ctDNA was extracted more frequently in higher-grade tumors, and the role of ctDNA detection in preoperative diagnosis is limited. The data suggest that ctDNA facilitates the accurate selection of patients at risk of severe disease who may require aggressive treatments.

In patients with co-occurring cancers with a high frequency of *BRAF* mutations, such as melanoma and carcinoma of the colorectal region, *BRAF^V600E^* positivity may not be diagnostic. A high level of ctDNA after definitive treatment is an important finding suggesting the need to search for other malignancies or other TC deposits.

New therapeutic options, such as molecular targeted agents, require appropriate diagnostic markers. The material obtained from the blood allows for real-time molecular assessment, providing information on genetic alterations acquired during the progression of the disease. ATC is characterized by areas of necrosis, which limits the molecular evaluation of biopsied tissue. However, determination of the molecular status in ATC is necessary for treatment selection, supporting the development of liquid biopsy methods for anaplastic carcinomas. Liquid biopsy methods may lead to a faster initiation of treatment because they provide a more efficient collection of material for testing. An important consideration is that the application of BRAFi therapy could potentially promote the uncontrolled growth of a clone lacking a targeted mutation, which may require sequential multigene testing. Detection of tumor growth in cases with negative liquid biopsy results obtained using single mutation typing should be an indication for liquid biopsy using NGS [72].

The *BRAF^V600E^* mutation affects the intake and metabolism of radioiodine in cancer cells, underscoring the importance of detecting this mutation in ctDNA for the selection of patients with RAIR TC who may benefit from BRAFi treatment, as data suggest that it might restore RAI uptake [73].

Active surveillance is a new option in the management of low-risk TC [4,5]. Although this approach may be advantageous in elderly patients and those with a high surgical risk, monitoring is limited to ultrasound evaluation as the presence of Tg is non-diagnostic in such patients. Additional information about the molecular status of the tumor may allow more personalized monitoring and is helpful for decisions regarding the discontinuation of conservative treatments.

The 2023 Bethesda System for Reporting Thyroid Cytopathology added ancillary molecular methods to the preoperative risk stratification of thyroid nodules with indeterminate FNAB results [74,75,76,77]. These methods are also limited by the risk of contamination from surrounding tissues with wild-type alleles [10]. Detection of *BRAF^V600E^* mutation in ctDNA shows lower sensitivity than FNAB molecular testing and is thus not an alternative to FNAB [4,78,79]. However, it may be useful in cases lacking lesion material for biopsy, or it can be used in combination to improve the assessment of disease status [46].

## 5. Conclusions and Future Directions

*BRAF^V600E^* detection in ctDNA is a low-risk test that holds great promise for advanced TC management, especially ATC. It may be particularly useful for the early detection of recurrence, the assessment of tumor heterogeneity and potentially actionable alterations, as well as the response or resistance to targeted therapy and the detection of molecular alterations in difficult-to-biopsy metastatic sites. NGS-based tests provide a broad perspective of the mutational status of a tumor and are thus helpful for selecting candidate mutations for further disease monitoring. A ddPCR provides reproducible results and is thus a useful tool for identifying select mutations for further evaluation of tumor progression. Careful selection of the appropriate assay for a particular group of patients is essential for the design of future studies.

## Figures and Tables

**Table 1:** Summary of currently used molecular methods.

Method		Limit of Detection	Turnaround Time	Estimated Cost per Reaction
Sanger sequencing		10% [19]	8 h	USD 100–200
PCR-based methods	qPCR	0.1% [20]	1–3 h	USD 200–300
	ddPCR	0.001% [21]	4–6 h	USD 200–500
NGS		2–15% [22]	1–13 days	USD 500–2000
NGS with molecular barcodes		0.2–0.01% [23,24]	1–2 weeks [25]	USD 1000–3000

PCR, polymerase chain reaction; qPCR, quantitative PCR; ddPCR, droplet digital PCR; NGS, next-generation sequencing.

**Table 2:** Summary of the results of methods for detecting *BRAF^V600E^* mutation in circulating tumor DNA in patients with thyroid cancer.

Method	Sensitivity		Specificity		Concordance (ctDNA vs. Tissue)	
	DTC	ATC	DTC	ATC	DTC	ATC
qPCR	0–91.7%	NA	30.0–100%	NA	23.9–89.5%	NA
ddPCR	31.0–47.6%	85.0%	80.0–100%	93.0%	31.0–62.7%	93.0%
NGS	0–4.5%	70.0–88.2%	100%	100%	41.7–60.0%	83.7–92.9%

ctDNA, circulating tumor DNA; DTC, differentiated thyroid cancer; ATC, anaplastic thyroid cancer; qPCR, quantitative PCR; NA, not available; ddPCR, droplet digital polymerase chain reaction; NGS, next-generation sequencing.

**Table 3:** Characteristics of the included studies.

Study (Year)	Number of Patients	Number of Patients with *BRAF* Positive TC	Pathological Type	Assay Type	Timepoint of Sampling	Concordance (ctDNA vs. Tissue)	Sensitivity	Specificity	Main Finding
Vdovichenko (2004) [28]	TC = 6	1	NA	PCR	Before the surgery	NA	NA	NA	Method failed to detect ctDNA.
Chuang (2009) [29]	28	5	PTC, FTC, BTL, Thyroid Lymphoma	Gap ligase chain reaction and PCR	Before the surgery	total 92.9%; 85.7% for PTC	60.0%	100.0%	*BRAF*-mutated ctDNA may be associated with more aggressive disease.
Cradic (2009) [30]	193	42	PTC, non-PTC	qPCR	During follow up for TC	53.3% for all TC; 37.5% for PTC	19.0%	97.0% for all TC; 93.0% for PTC	Presence of *BRAF*-mutated ctDNA was correlated with presence of active disease.
Pupilli (2013) [31]	168	12	PTC, TA, NNT, HC	qPCR	Before and after thyroid surgery	77.2%	91.7%	30.0%	Higher percentage of circulating *BRAF^V600E^* in PTC compared with BTL. Decrease in *BRAF^V600E^* ctDNA level after surgery.
Zane (2013) [32]	200	48	ATC, MTC, FTC, TA, PTC, HC	HRMA	After surgery	NA	NA	NA	Method failed to detect *BRAF*-mutated ctDNA.
Kwak (2013) [33]	94	94	PTC	qPCR	Before the surgery	NA	NA	NA	Method failed to detect ctDNA.
Fibbi (2014) [34]	1	1	MTC, PTC, and melanoma	qPCR	Before and after cancer treatments	NA	NA	NA	Decrease in ctDNA after cancer treatment.
Kim (2015) [35]	77	49	PTC, TA	qPCR	Before the surgery	40.3%	6.1%	100%	Positive *BRAF^V600E^* status in ctDNA associated with lung metastases.
Lubitz (2016) [36]	70	30	PTC, Hurthle cell neoplasm, BTL, FTC, MTC	RNA-based qPCR	Presurgical blood sample or during treatment of recurrent or metastatic PTC	71.0%	50.0%	86.8%	*BRAF* mutation in ctRNA is associated with a higher risk of LNM.
Janku (2016) [37]	TC = 10	8	NA	qPCR	Before initiation of BRAFi treatment and during therapy	70.0%	62.5%	100%	The changes in the ctDNA were similar to the changes in Tg concentration.
Konda (2017) [38]	20	20	RAIR DTC	ddPCR	During treatment with BRAFi + MEKi	35.0%	35.0%	NA	Detection of *BRAF^V600E^* mutation in ctDNA can be useful as indicator for treatment response.
Lupo (2017) [39]	66	NA	PTC, OTC, NIFTP	NGS	Before the FNAB	NA	NA	NA	Method failed to detect *BRAF^V600E^* mutated ctDNA.
Sandulanche (2017) [40]	23	10	ATC	NGS	At different stages of treatment	86.9%	70%	100%	Concordance of *BRAF^V600E^* detection in ctDNA and tissue was highest in treatment naïve patients.
Allin (2018) [41]	51	14	PTC, FTC, MTC, ATC, PDTC	ddPCR	After the surgery, at sequential timepoints	NA	NA	NA	The ctDNA was found in 67% of patients; may be superior in cases without a conventional marker and in assessing response to targeted therapies.
Condello (2018) [42]	83	22	PTC, BTL	ddPCR and qPCR	Before thyroid surgery	62.7%	0%	100%	Both methods failed to detect ctDNA.
Iyer (2018) [43]	44	20	ATC	ddPCR and NGS	Before surgery (*n* = 44), during treatment (*n* = 16)	93.0% for ddPCR; 91.0% for NGS	85.0% for ddPCR; 79.0%for NGS	100% for ddPCR and NGS	The ctDNA levels were predictive of treatment response. The ddPCR showed higher sensitivity and concordance than NGS in *BRAF^V600E^* detection in ctDNA.
Lubitz (2018) [44]	111	50	PTC	RNA-based qPCR	Before and after thyroid surgery or the initiation of treatment of advanced recurrent or metastatic PTC	For patients receiving initial surgery for PTC: 67.0%	For patients receiving initial surgery for PTC 64.0%	For patients receiving initial surgery for PTC: 72.0%	*BRAF*-mutated ctRNA was correlated with extrathyroidal extension. Decrease in *BRAF^V600E^*-mutated ctRNA after surgery and during adjuvant treatment.
Li (2019) [45]	59	26	PTC	dPCR	Before the surgery	77.9%	61.5%	90.91%	No association with clinical characteristics.
Jensen (2020) [46]	57	57	PTC	ddPCR with (COLD)PCR	Before the surgery	42.1%	42.1%	NA	Detection of *BRAF^V600E^*-mutated ctDNA is correlated with a higher risk of non-excellent response to primary treatment.
Khatami (2020) [47]	102	39	PTC	qPCR	Before the surgery	89.5%	84.6%	100%	*BRAF^V600E^*-mutated ctDNA correlated with LNM.
Wei (2020) [48]	10	4	PTC, TA	PCR with Sanger sequencing	Before the surgery	90.0%	100%	80.0%	*BRAF^V600E^*-mutated ctDNA was detected in benign lesions.
Cao (2020) [49]	20	8	PTC, TA	NGS	Before surgery	60.0%	0%	100%	Method failed to detect ctDNA.
Cabanillas (2020) [50]	3	4	PTC, ATC	NGS	Before initiation or during the BRAFi treatment	NA	NA	NA	Detection of *BRAF^V600E^* in ctDNA can be used to initiate therapy and to monitor disease progression.
Almubarak (2020) [51]	38	28	PTC	BEAMing and 3D dPCR, Sanger sequencing	During follow-up of patients with persisted disease or NED	NA	NA	NA	The ctDNA copy numbers were higher in metastatic than in non-metastatic disease. The ctDNA levels correlated with tumor burden.
Lan (2020) [52]	66	48, 22 with matched plasma	PTC	NGS	Before surgery	41.7%	4.5%	100%	Low sensitivity for *BRAF^V600E^* detection in plasma. *BRAF^V600E^* mutation was more common in locoregional tumors.
Suh (2021) [53]	127	41	PTC, FTC, NNT, BTL, HC	qPCR	Before thyroid surgery or during follow up	23.9%	0%	100%	Method failed to detect ctDNA.
Qin (2021) [54]	87	30	ATC	NGS	First plasma sample regardless of treatment status	92.9% for treatment naïve; 83.7% for previously treated	88.2%; NA	100%; NA	High concordance rate between tissue and ctDNA in treatment naïve patients. BRAFi therapy significantly increased OS.
Sato (2021) [55]	22	16	PTC	ddPCR	Before and after thyroid surgery	31.0%	31.0%	NA	Detection of mutated *BRAF^V600E^* in ctDNA indicates local progression of the primary tumor. Increase in mutated ctDNA after surgery predicts PTC recurrence.
Patel (2021) [56]	109	15	PTC, FTC	qPCR	Before and after thyroid surgery	40.0%	33.3%	60.0%	Detection of *BRAF^V600E^* in ctDNA correlated with higher staging and extrathyroidal extension. Decrease in circulating *BRAF* ctDNA after surgery.
Gouda (2022) [57]	33	30	PTC, FTC, PDTC	ddPCR	Before the surgery	NA	47.6%	80.0%	Patients with ctDNA *BRAF* WT had a shorter OS compared with patients with *BRAF^V600E^* detected in ctDNA.
Wei (2022) [58]	74	NA	PTC, TA	qPCR	Before surgery	73.1% (compared to FNAB)	NA	NA	High concordance rate between tissue and ctDNA in treatment naïve patients.
Dutta (2023) [59]	223	42	PTC, FTC, BTL	Allele-specific oligonucleotide PCR (8-gene panel)	Before surgery	NA	NA	NA	The ctDNA can be used as a marker of residual disease.
Tarasova (2023) [60]	1094	138	20% TC type reported (PTC, ATC, FTC, OTC, PDTC, MTC)	NGS	Retrospective analysis of the Guardant Health database	NA	NA	NA	*BRAF^V600E^* mutation was the second most common mutation in ctDNA, detected only in ATC and PTC.

TC, thyroid cancer; ctDNA, circulating tumor DNA; NA, not available; PCR, polymerase chain reaction; PTC, papillary thyroid cancer; FTC, follicular thyroid cancer; BTL, benign thyroid lesion; qPCR, quantitative polymerase chain reaction; TA, thyroid adenoma; NNT, non-nodular thyroid disease; HC, healthy controls; ATC, anaplastic thyroid cancer; MTC, medullary thyroid cancer; HRMA, high resolution melting assay; ctRNA, circulating tumor RNA; LNM, lymph node metastasis; BRAFi, BRAF inhibitors; Tg, thyroglobulin; RAIR, radioiodine refractory; ddPCR, droplet digital polymerase chain reaction; MEKi, MEK inhibitors; OTC, oncocytic thyroid cancer; NIFTP, non-invasive follicular thyroid neoplasm with papillary-like nuclear features; NGS, next-generation sequencing; FNAB, fine needle aspiration biopsy; PDTC, poorly differentiated thyroid cancer; dPCR, digital polymerase chain reaction; COLD PCR, co-amplification at lower denaturation temperature polymerase chain reaction; BEAMing, beads, emulsion, amplification, magnetics; NED, no evidence of disease; OS, overall survival; and WT, wild type.

## Data Availability

Not applicable.
